# Subgingival Microbiome in Pregnancy and a Potential Relationship to Early Term Birth

**DOI:** 10.3389/fcimb.2022.873683

**Published:** 2022-05-11

**Authors:** Irene Yang, Henry Claussen, Robert Adam Arthur, Vicki Stover Hertzberg, Nicolaas Geurs, Elizabeth J. Corwin, Anne L. Dunlop

**Affiliations:** ^1^ Nell Hodgson Woodruff School of Nursing, Emory University, Atlanta, GA, United States; ^2^ Emory Integrated Computational Core, Emory University, Atlanta, GA, United States; ^3^ Department of Periodontology, School of Dentistry, University of Alabama at Birmingham, Birmingham, AL, United States; ^4^ School of Nursing, Columbia University, New York, NY, United States; ^5^ Department of Gynecology and Obstetrics, School of Medicine, Emory University, Atlanta, GA, United States

**Keywords:** pregnancy, oral microbiome, periodontal disease, preterm birth, subgingival microbiome

## Abstract

**Background:**

Periodontal disease in pregnancy is considered a risk factor for adverse birth outcomes. Periodontal disease has a microbial etiology, however, the current state of knowledge about the subgingival microbiome in pregnancy is not well understood.

**Objective:**

To characterize the structure and diversity of the subgingival microbiome in early and late pregnancy and explore relationships between the subgingival microbiome and preterm birth among pregnant Black women.

**Methods:**

This longitudinal descriptive study used 16S rRNA sequencing to profile the subgingival microbiome of 59 Black women and describe microbial ecology using alpha and beta diversity metrics. We also compared microbiome features across early (8-14 weeks) and late (24-30 weeks) gestation overall and according to gestational age at birth outcomes (spontaneous preterm, spontaneous early term, full term).

**Results:**

In this sample of Black pregnant women, the top twenty bacterial taxa represented in the subgingival microbiome included a spectrum representative of various stages of biofilm progression leading to periodontal disease, including known periopathogens *Porphyromonas gingivalis* and *Tannerella forsythia.* Other organisms associated with periodontal disease reflected in the subgingival microbiome included several *Prevotella* spp., and *Campylobacter* spp. Measures of alpha or beta diversity did not distinguish the subgingival microbiome of women according to early/late gestation or full term/spontaneous preterm birth; however, alpha diversity differences in late pregnancy between women who spontaneously delivered early term and women who delivered full term were identified. Several taxa were also identified as being differentially abundant according to early/late gestation, and full term/spontaneous early term births.

**Conclusions:**

Although the composition of the subgingival microbiome is shifted toward complexes associated with periodontal disease, the diversity of the microbiome remains stable throughout pregnancy. Several taxa were identified as being associated with spontaneous early term birth. Two, in particular, are promising targets of further investigation. Depletion of the oral commensal *Lautropia mirabilis* in early pregnancy and elevated levels of *Prevotella melaninogenica* in late pregnancy were both associated with spontaneous early term birth.

## 1 Introduction

Pregnant women are susceptible to periodontal disease (Raju and Berens, 2021) due to hormonally driven hyper-reactivity of the gingiva to bacteria in the subgingival biofilm (Usin et al., 2013). Other factors that increase risk for poor oral health during pregnancy include changes in dietary habits (frequent snacking or increased consumption of carbohydrate rich or decay-promoting foods), stomach acids from nausea and vomiting that contribute to the breakdown of tooth enamel, and decreased likelihood of seeking dental care during pregnancy (Detman et al., 2010).

Periodontal disease can be understood as two sub-conditions. Gingivitis, the milder form of periodontal disease, occurs in 50-70% of all pregnancies (Anil et al., 2015). Gingivitis is an inflammation of the gingiva in which the connective tissue attachment to the tooth remains intact. The inflammation is limited to the soft-tissue compartment of the epithelium and connective tissue (Beck and Arbes, 2006). Symptoms typically emerge during the first trimester (Giglio et al., 2009) and present as red, swollen gingival margins and bleeding that occurs with the slightest provocation (Barak et al., 2003). The severity of gingivitis increases as pregnancy progresses (Cohen et al., 1969). Progressive gingivitis can lead to periodontitis, a more severe and chronic form of periodontal disease involving the irreversible destruction of supportive soft tissue and bone, ultimately leading to tooth loss (Darby et al., 2000). Diagnosis of periodontitis is based on clinical measurements of subgingival pocket depth, bleeding on probing, a plaque index, clinical attachment level, and radiographic examination (Papapanou et al., 2018). Symptoms of periodontal disease typically become more overt during the second trimester (American Pregnancy Association, 2014) in approximately 5-20% of pregnant women (Jared and Boggess, 2008), however, oral assessment, a basic component of any physical exam, is frequently overlooked in prenatal care. Dental care utilization in pregnancy ranges from 25% to 75% and is highly associated with demographic, socioeconomic, and perceived need factors (Rocha et al., 2018).

Decades of epidemiological research suggest that periodontal disease is an independent risk factor for various adverse birth outcomes, including preterm birth (Komine-Aizawa et al., 2019). Since 1996 when Offenbacher et al. (1996) reported that women with periodontal disease had a seven-fold increase in the risk of preterm birth, results from several clinical studies have confirmed this association. For example, after controlling for other known risk factors of preterm birth such as systemic disease, smoking, or complications during previous pregnancies, moderate to strong associations were found between periodontal disease and/or inflammation and spontaneous preterm birth (Khader et al., 2009; Guimarães et al., 2010; Macedo et al., 2014; Stadelmann et al., 2014). These associations, however, must be considered in light of a high degree of variability in study populations, and clinical assessment (Agueda et al., 2008; Ide and Papapanou, 2013) informing our choice to focus our investigation on a within-race sample of African American women, who are at greater risk for both preterm birth and periodontal disease (Henshaw et al., 2018; Martin et al., 2018).

Periodontal disease is initiated by microbial dysbiosis. The human oral cavity has a characteristic microbiome, with over 700 bacterial species (Aas et al., 2005), representing the second most complex ecosystem in the body, after the colon (Huttenhower et al., 2012). The key to oral health is an ecologically balanced and diverse oral microbiome that is in a state of commensalism within itself and mutualism with its host (Zaura et al., 2009; Zarco et al., 2012). This balanced biodiversity is mutually beneficial for both host and microbial community. Acute disruptions like pregnancy can challenge a stable ecosystem, increasing the risk of infection by opportunistic pathogens (Wade, 2013). Persistent and prolonged disruptions can permanently shift the composition of a microbial community. Overgrowth of opportunistic pathogens may lead to a cascade of interactions between host and community, which over time, lead to the development of a new community. This new community is equally as stable, but reflective of a diseased state (Socransky and Haffajee, 2005). Local oral pathogenesis is now accepted as an ecological phenomenon. Dysbiosis within an ecological niche plays a role in all major oral disease including periodontal disease (Dewhirst et al., 2010) shifting biodiversity to initiate an infectious/inflammatory state (Zarco et al., 2012). The microbial etiology of periodontal disease suggests that a thorough understanding of the microbiome is essential for any investigation of the association between periodontal disease and preterm birth.

The prenatal oral microbiome, however, is not well understood. Early attempts to characterize the ecological shift that occurs in pregnancy were limited to the identification of specific pathogenic organisms like *Porphyromonas gingivalis, Treponema denticola*, and *Tannerella forsythia* (Holt and Ebersole, 2005). Results were inconsistent (Jarjoura et al., 2005; Lin et al., 2007; Novak et al., 2008; Vettore et al., 2008; Adriaens et al., 2009; Carrillo‐de‐Albornoz et al., 2010) due to the methodologies employed in studies which only targeted known pathogens. Emerging periodontal disease research utilizing 16S rRNA next-generation sequencing in the non-pregnant population implicate many more associated taxa and uncultivated species in the subgingival biofilm (Griffen et al., 2012; Liu et al., 2012) that have not been investigated in the prenatal population. These include genera more abundant in periodontal disease like *Spirochaetes*, *Synergistetes, Bacteriodetes, Clostridia, Negativicutes*, and *Erysipelotrichia* (Griffen et al., 2012), *Selenomonas, Prevotella, Treponema, Tannerella, Haemophilus*, and *Catonella* (Liu et al., 2012), whereas, *Bacilli, Proteobacteria* (Griffen et al., 2012)*, Streptococcus, Actinomyces*, and *Granulicatella* (Liu et al., 2012) were associated with healthy gingiva. Furthermore, this shift in the relative abundance of microbial organisms associated with oral health and disease appears to be present even in early and mild cases, i.e., gingivitis, where frank periodontitis is not yet evident (Liu et al., 2012). This ecological shift, however, is not yet fully understood for periodontal disease in general, and for pregnant women, in particular.

The purpose of this study, therefore, was to characterize the structure and diversity of the subgingival microbiome of African American women in early and late pregnancy and explore relationships between the microbiome and preterm birth.

## 2 Materials and Methods

### 2.1 Design

This study utilized a longitudinal design with a sample of 59 participants drawn from among a larger group of participants taking part in the Emory University African American Vaginal, Oral, and Gut microbiome in Pregnancy cohort study. Institutional review board approval was obtained for this study the primary purpose of which was to characterize the subgingival microbiome at two points during pregnancy: early gestation (8-14 weeks) and late gestation (24-30 weeks).

### 2.2 Setting and Sample

Participants for this study were recruited from an ongoing investigation of the association between a woman’s microbiome and preterm birth (Corwin et al., 2017). The parent study prospectively enrolled a socioeconomically diverse cohort of pregnant women between 8-14 weeks gestation who were US born Black or African American and followed them through delivery. Participants who were enrolled in the parent study were approached at the initial data collection period within the 8-14 week gestation period to ascertain their interest in and eligibility for this study. Additional eligibility criteria for this study included having: 1) minimum of 20 natural teeth and 2) no dental cleaning in the past three months. These requirements are routinely used in oral health studies (Eick and Pfister, 2002; Griffen et al., 2012; Matthews et al., 2013) to ensure adequate oral sampling sites and an undisturbed subgingival environment. Use of antibiotics was collected to address potential confounding effects on the microbiome, however, none of the participants whose samples were included in our microbiome analyses reported recent use of antibiotics.

### 2.3 Procedures

#### 2.3.1 Oral Microbiome Samples

Subgingival plaque samples were collected for microbiome analysis using protocols based on the Human Microbiome Project (McInnes and Cutting, 2010). Participants were asked about oral sex within 48 hours of sample collection (McInnes and Cutting, 2010), oral hygiene (tooth brushing, flossing, and mouth washes) within 12 hours of sample collection (Kumar et al., 2014), and food, drink or gum within 30 minutes of sample collection (Kumar et al., 2014) as potential confounders of microbiome analysis. Samples were collected in early pregnancy (8-14 weeks) and again in late pregnancy (24-30 weeks).

Subgingival plaque was collected by inserting a scaler into the gingival sulcus of three tooth sites exhibiting visible signs of inflammation (McInnes and Cutting, 2010). If no visible signs of inflammation were present, three tooth sites were chosen randomly. After collection, each scaler tip was immediately swirled and placed in 750uL of MoBio buffer contained in sterile MoBio bead tubes (Mobio laboratories, Inc., Carlsbad, CA). These tubes were then placed on ice in a biohazards transport bag and transported for storage at -80°C until ready for DNA extraction.

#### 2.3.2 DNA Isolation and 16S rRNA Library Preparation and Sequencing

Specimens were sent to the Emory Integrated Genomics Core (Atlanta, GA). DNA was isolated using the Qiagen DNeasy Powersoil Kit (Qiagen; 12888). Libraries were made using a modification of the Illumina 16S Meta-genomic Sequencing Library Preparation workflow (Illumina Inc., 2020). Briefly, the highly conserved 16S rRNA gene, which is widely used to characterize taxonomic diversity in microbial communities, was amplified targeting the third and fourth hypervariable region (v3-v4). Final 16S libraries were approximately 630 base pairs (bp) in length and were pooled in equal amounts based on fluorescence quantification. Final library pools were quantitated *via* quantitative Polymerase Chain Reaction (qPCR) (Kapa Biosystems; KK4824). The pooled library was sequenced on an Illumina MiSeq using MiSeq v3 600 cycle chemistry (Illumina; MS-102-3003) at a loading density of 6–8 pM with 20% PhiX, generating roughly 20 million, 300 bp paired-end reads.

#### 2.3.3 Quality Control and Amplicon Bioinformatics

All samples were collected using standard sterile technique. Consistent reagents were used throughout DNA extraction with positive control (*Escherichia coli* bacterial pellet) to ensure appropriate extraction and negative control (sterile water) to confirm no contamination in extraction kit reagent. Additional controls (positive: mock community with known microbiome diversity; negative: sterile water) were used in the PCR amplification process.

Amplicon sequence reads in compressed fastq.gz format were produced by the above extraction and subsequent sequencing protocols and were then checked for quality control with FastQC and MultiQC packages as seen in Yang et al., 2021 (Andrews, 2010; Ewels et al., 2016). Following, reads were analyzed *via* Quantitative Insights Into Microbial Ecology (QIIME2) 2020.2 (Bolyen et al., 2019). Data denoising and dereplication was implemented by use of the DADA2 module in QIIME2 (Callahan et al., 2016), and the amplicon sequencing variant (ASV) feature table was built. DADA2 read trimming and truncation parameters of trim-left-f and trim-left-r 30 and trunc-len-f and trunc-len-r 240 were used. Taxonomic assignment using the v3-v4 hypervariable regions of the 16S gene for primers was conducted, and data were compared to GreenGenes (v13_8, 99% clustered OTUs) (DeSantis et al., 2006), Silva v132 (Quast et al., 2012), and the Human Oral Microbiome Database (HOMD) v15.2 (Chen et al., 2010) through QIIME2 taxonomy modules (Bolyen et al., 2019).

In further detail, reference reads were pulled *via* QIIME2 feature-classifier extract-reads, trimming as above to match the raw reads using v3/v4 primers, then fitted to a naïve-Bayesian classifier using QIIME2 feature-classifier fit-classifier-naïve-bayes, and applied to the ASV feature table using QIIME2 feature-classifier sklearn (Bolyen et al., 2019). The HOMD database best resolved our Zymo microbial mock community controls and positive controls, thus HOMD taxonomic assignment was used downstream. Data were then exported into.BIOM format for downstream analyses.

#### 2.3.4 Clinical and Questionnaire Data

Demographic and health behavior variables were collected from parent study survey data and birth outcome data were collected from parent study medical record abstraction reports. Gestational age at birth outcomes were adjudicated to include the full range of classifications as identified in the American College of Obstetrics and Gynecology (ACOG) guidelines. Preterm births, identified as birth prior to 37 weeks gestation (American College of Obstetricians and Gynecologists, 2013), were grouped according to whether they were spontaneous or induced. Births after 37 weeks were categorized as “early term” from 37 0/7 weeks through 38 6/7 weeks, and full term from 37 0/7 weeks through 38 6/7 weeks (American College of Obstetricians and Gynecologists, 2013). Spontaneous abortions (loss of pregnancy at less than 20 weeks gestation) (American College of Obstetricians and Gynecologists' Committee on Practice Bulletins—Gynecology, 2018) were also included as a group. All participants received early pregnancy dating by last menstrual period (LMP) and/or early ultrasound, given enrollment criteria. Type of Labor (spontaneous, induced, none) and mode of delivery (vaginal, C-section) along with indication for induction and/or C-section were obtained and used to further phenotype birth outcomes.

#### 2.3.5 Analysis

From a total of 106 samples (across both time points), we excluded 34 samples due to low yield, specifically, any samples with less than 20,000 reads remaining after DADA2 dereplication and filtering were removed. As a result, the final study sample consisted of 72 subgingival samples (38 early pregnancy samples and 34 late pregnancy samples) from 50 participants. Sociodemographic and self-reported oral symptom and behavior data for these 50 participants were described. Analysis comparing the subgingival microbiome at early and late gestation overall was conducted among samples, regardless of birth outcome (*N* [Time 1] = 38; *N* [Time 2] = 34). Analysis comparing the subgingival microbiome according to gestational age at birth outcomes excluded inductions and included spontaneous abortions and spontaneous births (preterm, early term, and full term). Comparative analyses were conducted with the full term group as the referent group, and were conducted both at early and late gestation.

Alpha diversity, a measure of species diversity within a particular community, was calculated using the Shannon index within the QIIME2 platform. The Shannon index provides a measure of both richness and evenness (Magurran, 2013). Communities numerically dominated by one or a few species exhibit a low Shannon score, whereas communities in which abundance is distributed equally among species will exhibit high evenness. Beta diversity, which measures similarity/dissimilarity between a pairs of communities, were calculated using the Bray-Curtis (abundance-weighted) and Jaccard (presence/absence of detected ASV) distances. The communities were visualized on a principal coordinates analysis (PCoA) plot based on these distance matrices to assess any clustering by groups of interest. The significance of the cluster differences (i.e., variation in community structure in relationship to group status) was assessed using the permutational multivariate analysis of variance (PERMANOVA).

The Linear Decomposition Model (LDM) (Hu and Satten, 2020) was used to determine differences at the individual ASV level with permutation-based *p*-values, in terms of relative abundance, controlling for false discovery rate at a nominal level of 5%. Because this is an exploratory study, significant *p*-values, even though differences did not persist with correction, were reported as potential signals for future targeted investigation. Sample groups were classified by spontaneous pre-term, spontaneous early term, or full-term gestation. Taxa, by relative abundance, were tested for differences using LDM’s global test of the microbiome effect. To account for the difference in sample size, random sampling with replacement was implemented to match sample group sizes and the global test repeated for 1,000 permutations.

## 3 Results

### 3.1 Sociodemographic Characteristics and Birth Outcomes

The mean age of the participants was 26.04 ± 5.37 years. Other sociodemographic characteristics and birth outcomes may be found in **Table 1**.

**Table 1:** Sociodemographic characteristics of participants according to birth outcomes (*N* = 50).

Sociodemographic Characteristics	Preterm (*n* = 5)	Early Term (*n* = 16)	Full Term (*n* = 24)	Spontaneous Abortions (*n* = 3)
Education				
≤ High School	4 (80.0)	9 (56.25)	13 (54.2)	2 (0.67)
> High School	1 (20.0)	3 (18.75)	9 (37.5)	1 (0.33)
Missing	0	4 (2.5)	2 (8.3)	0
Income				
< 100% Federal Poverty Level	2 (40.0)	9 (56.25)	9 (37.5)	3 (100)
≥ 100% Federal Poverty Level	3 (60.0)	2 (12.5)	8 (33.3)	0
Missing	0	5 (31.25)	7 (29.2)	0
Medical Insurance				
Medicaid	4 (80.0)	11 (68.75)	20 (83.4)	3 (100)
Private Insurance	1 (20.0)	1 (6.25)	2 (8.3)	0
Missing	0	4 (25.0)	2 (8.3)	0

Twin birth (n = 1); Missing sociodemographic data (n = 1).

### 3.2 Self-reported Oral Healthy Symptom and Behavior

Only one participant self-reported gingivitis at the second time point. Other oral health symptoms and behaviors are in **Table 2**.

**Table 2:** Self-reported oral health symptoms and behaviors in early and late gestation.

	Early Gestation (*n* = 38)	Late Gestation (*n* = 34)
	Frequency, *n* (%)	Frequency, *n* (%)
*In the past month*		
Self-reported gingivitis		
Yes	0	1 (2.9)
No	38 (100)	33 (97.1)
Missing	0	1 (2.9)
Used mouthwash		
Yes	24 (63.2)	19 (55.9)
No	13 (34.2)	14 (41.2)
Missing	1 (2.6)	1 (2.9)
Flossed		
Yes	23 (60.5)	12 (35.3)
No	14 (36.8)	21 (61.8)
Missing	1 (2.6)	1 (2.9)
Visited dentist		
Yes	2 (5.3)	3 (8.8)
No	35 (92.1)	31 (91.2)
Missing	1 (2.6)	0
*Current*		
Red and swollen gums		
Yes	1 (2.6)	1 (2.9)
No	37 (97.4)	33 (97.1)
Bleeding gums		
Yes	11 (28.9)	8 (23.5)
No	26 (68.4)	25 (73.5)
Missing	1 (2.6)	1 (2.9)
Brushed teeth in the last 2 days		
Yes	37 (97.4)	32 (94.1)
No	0	1 (2.9)
Missing	1 (2.6)	1 (2.9)

### 3.3 Characterization of the Oral Microbiome Across Early and Late Pregnancy

**Figure 1:**
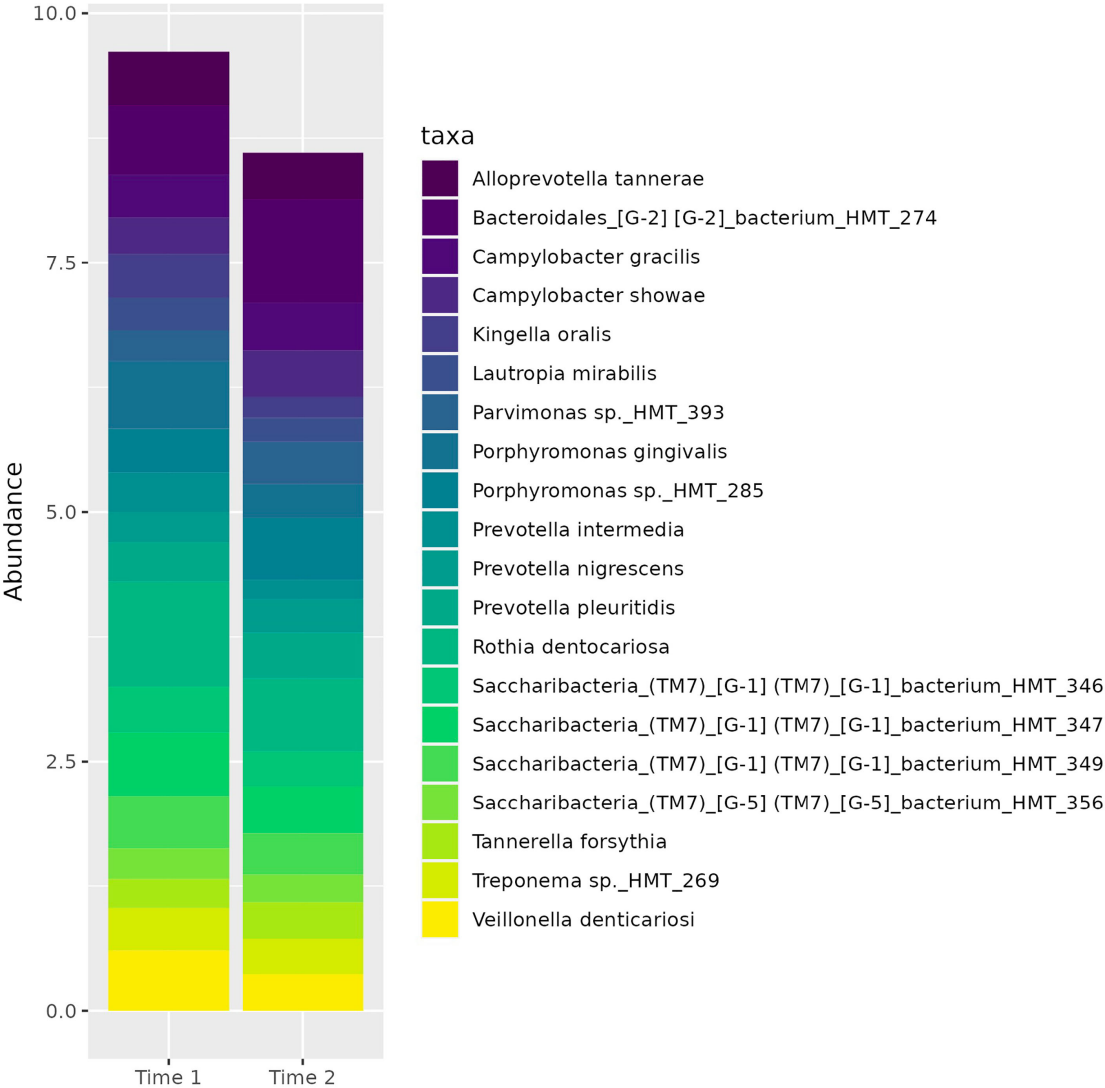
Top 20 species (according to relative abundance) across Time 1 and Time 2.

There was no difference in measures of alpha or beta diversity for samples from early and late gestation, controlling for participants. The top twenty taxa (**Figure 1**) represented in the subgingival microbiome of participants across gestation include a spectrum of bacteria representative of various stages of biofilm progression leading to periodontal disease. These include *Porphyromonas gingivalis* and *Tannerella forsythia.* Other bacteria associated with periodontal disease that were reflected in the microbiome profile of our participants included several *Prevotella* spp., and *Campylobacter* spp.

The relative abundance of six taxa differentiated the subgingival microbiome of our cohort between early and late pregnancy. Five organisms (*Porphyromonas* sp.*_HMT_284, Catonella* sp.*_HMT_164, Peptostreptococcaceae_[XI][G-5] [XI][G-5]_saphenum, Johnsonella ignava, Actinomyces massiliensis* were more abundant in early pregnancy, while one, *Cryptobacterium curtum*, was more abundant in late pregnancy (**Table 3**).

**Table 3:** Six taxa identified to have significantly different relative abundances between early and late pregnancy.

Taxa	Early Pregnancy Mean Relative Abundance	Late Pregnancy Mean Relative Abundance	*p.*value	fdr.*q*.value
*Actinomyces massiliensis*	0.0311	0.01313	0.0317	0.996
*Catonella* sp.*_HMT_164*	0.0185	0.01083	0.0078	0.7271
*Cryptobacterium curtum*	0.00227	0.01102	0.0127	0.7271
*Johnsonella ignava*	0.00851	0.00316	0.0196	0.8977
*Peptostreptococcaceae_[XI][G-5] [XI][G-5]_saphenum*	0.00314	0.00225	0.0108	0.7271
*Porphyromonas* sp.*_HMT_284*	0.00514	0.0051	0.0034	0.7271

### 3.4 Characterization of the Oral Microbiome According to Birth Outcomes

**Figure 2:**
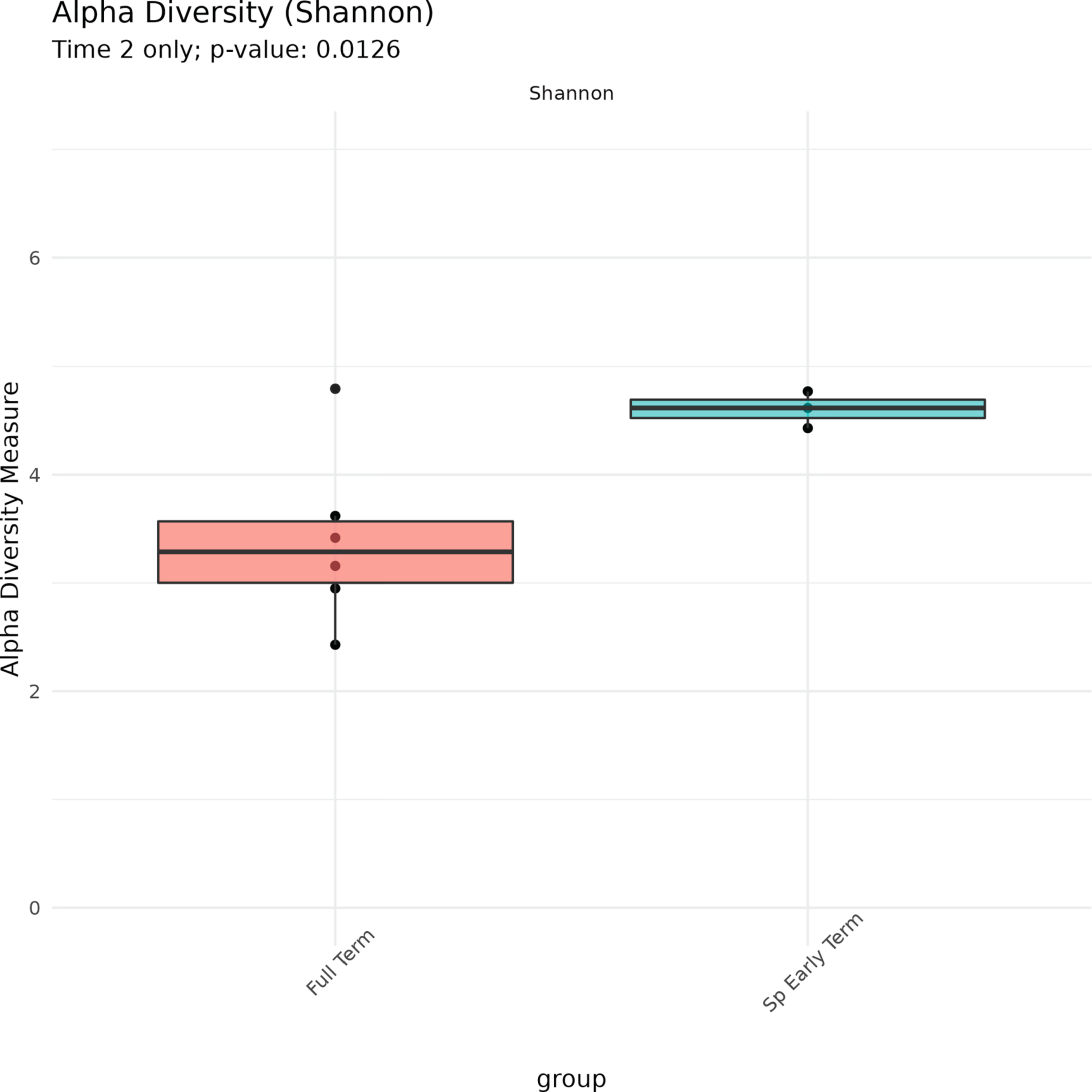
Alpha diversity of subgingival microbiome at Time 2 according to delivery time status.

**Figure 3:**
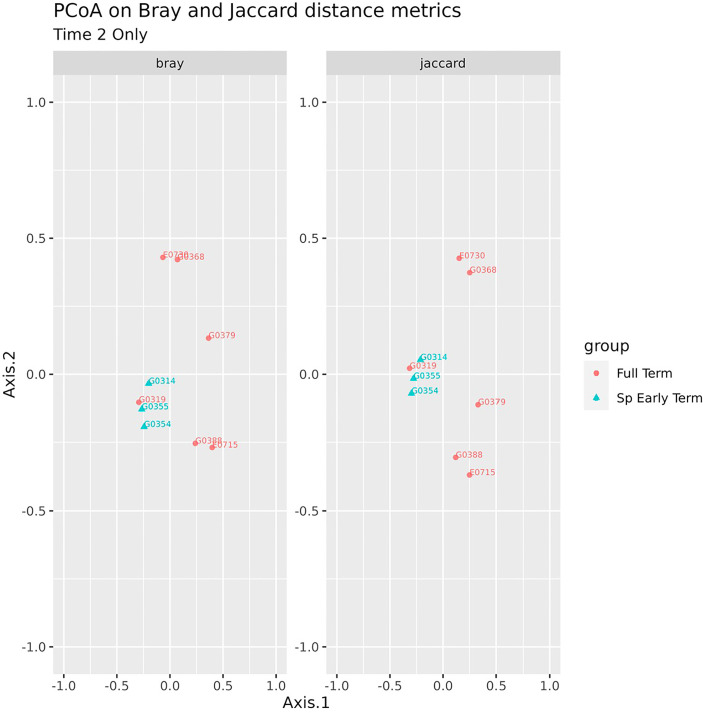
Beta diversity of subgingival microbiome at Time 2 according to delivery time status.

No association between microbiome features and spontaneous abortion or spontaneous preterm birth at either time point were identified. We did, however, find that in late pregnancy both alpha and beta diversity distinguished the subgingival microbiome of women who spontaneously delivered early term from women who delivered full term (**Figures 2**, **3**). Several taxa also differentiated women who spontaneously delivered early term vs full term. In early pregnancy, eight taxa including *Actinomyces israelii, Cardiobacterium hominis, Treponema putidum*, and *Lautropia mirabilis* were more abundant among women who delivered full term compared to early term (**Table 4**). In late pregnancy, four taxa (*Campylobacter gracilis, Prevotella melaninogenica*, and two unnamed species belonging to the genera *Tannerella* and *Catonella*) were differentially abundant between women who spontaneously delivered early term and women who delivered full term. Of these, *P. melaninogenica* was the only organism that was more abundant among women who spontaneously delivered early term (**Table 5**).

**Table 4:** Eight taxa identified in early pregnancy to have significantly different relative abundances between spontaneous early term and full term births.

Taxa	Spontaneous Early Term Mean Relative Abundance	Full Term Mean Relative Abundance	*p*.value	fdr.*q*.value
*Actinomyces israelii*	0.00118	0.00132	0.003	0.2332
*Leptotrichia* sp.*_HMT_392*	0.00158	0.00279	0.0047	0.2332
*Cardiobacterium hominis*	0.00515	0.0077	0.0076	0.2332
*Capnocytophaga* sp.*_HMT_338*	0.00023	0.00216	0.0093	0.2332
*Treponema putidum*	0.00318	0.00765	0.0106	0.2332
*Bacteroidaceae_[G-1] [G-1]_bacterium_HMT_272*	0.00085	0.00289	0.0166	0.3043
*Lautropia mirabilis*	0.00428	0.01146	0.0419	0.6435
*Prevotella loescheii*	0.00024	0.00795	0.0468	0.6435

**Table 5:** Four taxa identified in late pregnancy to have significantly different relative abundances between spontaneous early term and full term births.

Taxa	Early Term Mean Relative Abundance	Full Term Mean Relative Abundance	*p*.value	fdr.*q*.value
*Campylobacter gracilis*	0.00838	0.03764	0.025	0.3132
*Catonella* sp.*_HMT_164*	0.00132	0.00364	0.0464	0.3132
*Prevotella melaninogenica*	0.01081	0.00366	0.0435	0.3132
*Tannerella* sp.*_HMT_286*	0.00211	0.00512	0.0452	0.3132

## 4 Discussion

### 4.1 Sociodemographic and Behavioral Characteristics

This study describes the subgingival microbiome of a group of pregnant Black women and explores associations between the microbiome and preterm birth. Although the parent study, from which this study leveraged its participants, aimed to enroll a socioeconomically diverse cohort of pregnant women, the majority of our convenience sub-sample were socioeconomically vulnerable with less than high school education, and incomes at less than the federal poverty level. Most of the participants were on Medicaid insurance. Evidence suggests that socioeconomic inequalities are strongly associated with oral health disparities, both self-reported and clinically determined (Mejia et al., 2018). This holds true for pregnant populations as well (Azofeifa et al., 2014; Chung et al., 2014; McNeil et al., 2016). None of the women in the study self-reported gingivitis across pregnancy, although one woman reported red or swollen gums, and around a quarter of the participants reported bleeding gums across pregnancy. Both red/swollen gums and bleeding gums are symptoms of gingivitis. Self-reported gingivitis and symptomatology is less than expected within our cohort since we know that gingivitis is present in 50-70% of pregnant women (Anil et al., 2015), and tends to increase in severity across pregnancy (Cohen et al., 1969). Self-reported gingivitis and bleeding gums, while useful for public health screening, lacks validity for individual screening for gingivitis as defined by a dental professional (Kallio et al., 1994; Abbood et al., 2016). It is likely that underreporting of gingivitis and associated symptoms was present in our study.

Virtually all the women reported brushing their teeth in the last two days, however, use of other oral hygiene measures such as flossing, and mouthwash were not as ubiquitous. This can be understood in the light of previous research which indicates racial, ethnic, and economic disparities related to oral hygiene practices during pregnancy (Boggess et al., 2010). Even controlling for income, education, and insurance status, racial disparities in oral health experiences persist; an important contextual consideration for our cohort (Hwang et al., 2011).

### 4.2 The Subgingival Microbiome Across Pregnancy

Microbial community diversity can be described in two ways. Alpha diversity describes how many ASVs are present in a community and how evenly they are distributed; beta diversity describes the diversity between two different environments. In studies conducted among an Asian population, pregnancy itself appears to increase the alpha diversity of the subgingival microbiome (Balan et al., 2021), although both species richness and diversity remain stable throughout pregnancy (Balan et al., 2018). Our findings support the consistency of alpha diversity throughout pregnancy among African American women since we found no difference in either species richness or diversity between early and late pregnancy. More research among diverse racial and ethnic populations of pregnant women is needed to further assess the generalizability of this finding.

**Figure 4:**
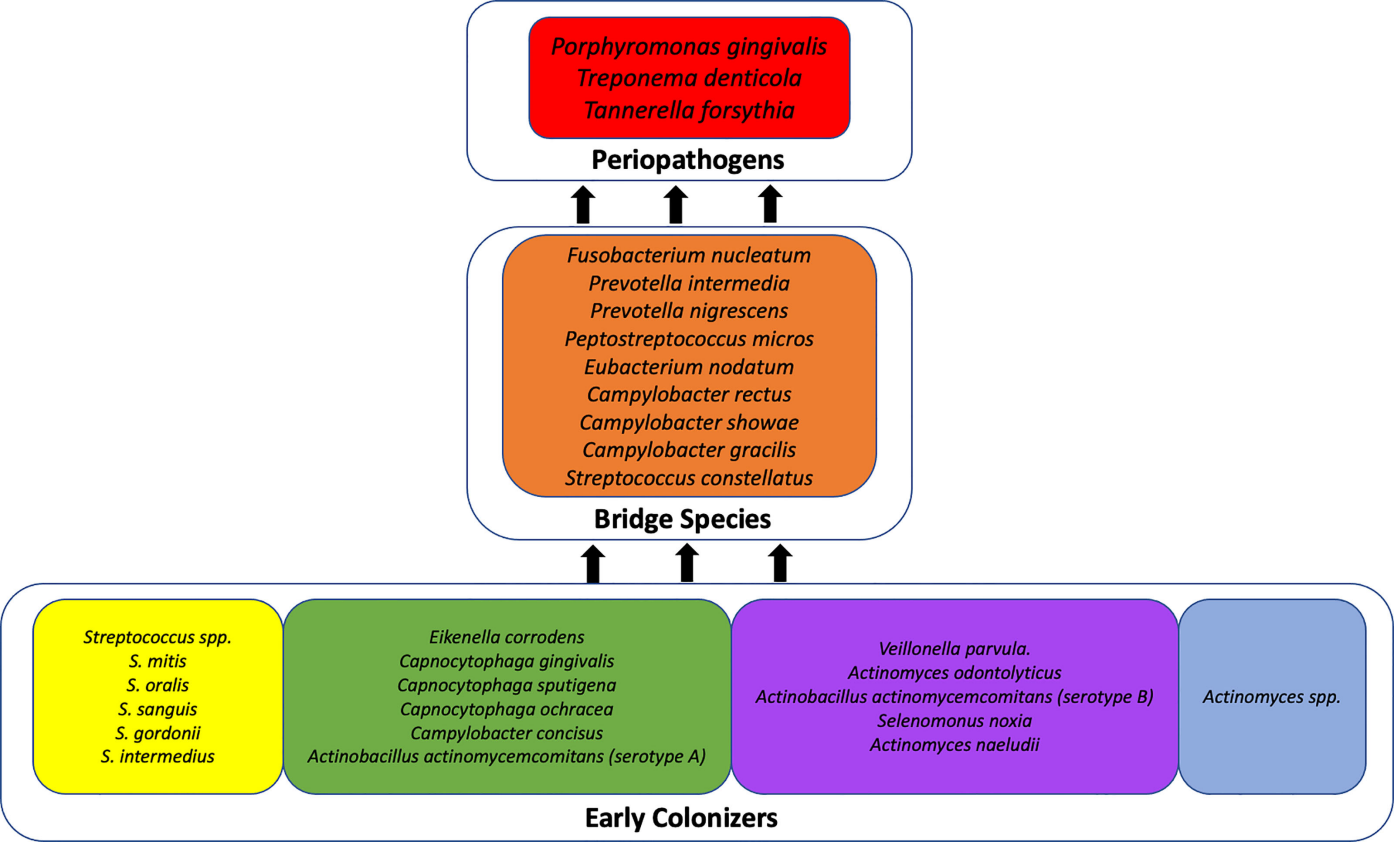
Theoretical scheme of microbial succession in periodontal disease. This figure has been adapted from Socransky and Haffajee, 2005; Publisher: Wiley; Copyright © Blackwell Munksgaard 2005.

To contextualize our description of the most prevalent organisms identified in the subgingival space of our cohort of pregnant women, it is important to understand that periodontal disease has a polymicrobial etiology. The current scientific understanding of periodontal disease is that of progressive dysbiosis within the biofilm, a polymicrobial ecosystem which attaches to the surface of the tooth in the subgingival pocket (Guthmiller and Novak, 2002). The dysbiotic shift results in a predominantly gram negative environment (Mohanty et al., 2019) caused by microbial succession that occurs with rapid accumulation of plaque on the teeth (Socransky and Haffajee, 2005). According to the seminal work of Socransky and Haffajee (2005), the postulated scheme of microbial succession occurs according to complexes, or co-occurring clusters of organisms. Initially, members of the yellow, green, and purple complexes dominate along with *Actinomyces* species (Socransky and Haffajee, 2005). The establishment of these three complexes contributes to the alteration of the subgingival environment such that the next two, more pathogenic, complexes become dominant (**Figure 4**) (Socransky and Haffajee, 2005). These are the “orange complex” organisms (*Prevotella intermedia, Prevotella nigrescens, Peptostreptococcus micros, Fusobacterium nucleatum, Eubacterium nodatum, Streptococcus constellatus*, and several *Campylobacter* species). The orange complex of organisms contain bridge species and generally precedes the growth of the red complex which consists of *Treponema denticola, Porphyromonas gingivalis*, and *Tannerella forsythia* (Guthmiller and Novak, 2002). The red complex organisms are known as periopathogens and are present at sites presenting with symptoms of periodontal disease.

Notably, several of the organisms represented among the top 20 most abundant organisms in the subgingival microbiome of our cohort include members of both the red and orange complexes: *Porphyromonas gingivalis, Prevotella intermedia, Prevotella nigrescens, Campylobacter gracilis*, and *Campylobacter showae*, suggesting a shift toward a pathogenic subgingival ecosystem. This finding is supported by a previous study by Balan et al. (2018) which also identified a dominance of periopathogens during pregnancy from the genera *Prevotella, Veillonella*, and *Streptococcus*, and at the species level, *P. gingivalis, P. nigrescens*, and *P. oris.*

Given that periodontal disease symptoms intensify over pregnancy (Cohen et al., 1969), we might expect to see increases in the abundance of these pathogenic organisms over pregnancy, however, the significant changes in relative abundance of organisms that we identified were not among established periodontal disease associated organisms. Instead, slight but significant differences were identified with six taxa, five of which decreased from early to late pregnancy, and one which increased in abundance across pregnancy. Of the five taxa that decreased in abundance, three were uncultivated phylotypes. Little is known about *Porphyromonas* sp.*_HMT_284*, however, sister organisms within the genus are *P. gingivalis* and *P. endodontalis*, both of which have been associated with periodontal disease (Lombardo Bedran et al., 2012). The genus *Catonella* is a relatively newly identified genus (Moore and Moore, 1994) with one named species, *C. morbi*, that has been described as a putative periopathogens (Wu et al., 2017). Bacteria from the family *Peptostreptococcaceae* are a morphologically diverse group of gram positive organisms (Slobodkin, 2014). Due to the multiple genera and species within this family it is difficult to speculate on the meaning of our finding that this particular taxa decreased in abundance over pregnancy. *Johnsonella ignava* may be an opportunistic pathogen. There is modest evidence that it may be associated with chronic obstructive pulmonary disease as well as oral squamous-cell carcinoma (Wu et al., 2017). The evidence on whether it is associated with periodontal disease is mixed (Moore and Moore, 1994; Wu et al., 2017). *Actinomyces* spp. are commonly found in human oral mucosa and many novel species have been described in recent years (Renvoise et al., 2009). Although *A. massiliensis* is known to reside in the subgingival biofilm, its role within an *Actinomyces* complex associated with periodontal disease is not known (Vielkind et al., 2015). *Cryptobacterium curtum* was the only taxa identified to increase from early to late pregnancy. *Cryptobacterium curtum* is an oral opportunistic pathogen that is involved in dental and oral infections (Mavrommatis et al., 2009).

Our findings suggest that despite the overall stability in alpha and beta diversity over the course of pregnancy, relative abundance shifts within individual taxa do occur over time with organisms that have some associations to periodontal disease and other opportunistic infections. Further investigation of these organisms and their clinical impact on oral disease and pregnancy outcomes are warranted.

### 4.3 Exploring the Association Between the Subgingival Microbiome and Preterm/Early Term Birth

Likely due to limited power from the small number of preterm births in our cohort, we did not identify any associations between subgingival microbiome features and spontaneous preterm birth. We did, however, identify associations among microbiome features and spontaneous early term birth (37 0/7 weeks through 38 6/7 weeks). During early pregnancy, eight taxa were more abundant among women who delivered full term compared to those who delivered early term. Two were unnamed species belonging to the genera *Leptotrichia* and *Capnocytophaga. Leptotrichia* spp. are facultative anaerobic oral commensals that are commonly found in the oral cavity (Smid et al., 2015) and can act as opportunistic pathogens or stimulate the growth of other pathogenic organisms (Eribe and Olsen, 2017). Various pathologic conditions are associated with *Leptotrichia*, (Eribe and Olsen, 2017) including poor pregnancy outcomes. *Leptotrichia amnionii*, *L. sanguinegens*, and *L. buccalis* have been associated with outcomes such as miscarriage, chorioamnionitis, preterm labor, pregnancy loss, neonatal infection and postpartum infection (Smid et al., 2015). Several *Capnocytophaga* spp. belong to the green complex, early colonizers in the microbial succession leading to periodontal disease.

*Cardiobacterium hominis* was also more abundant among women who delivered full term. *C. hominis* is not commonly associated with oral disease, although one study did find it to be associated with aggressive periodontitis and suggested it as a potentially new periopathogens (Lourenço et al., 2014). *C. hominis* is better known as an oral commensal belonging to the HACEK group of five organisms which can cause infective endocarditis (Moreillon, 2010).

Oral treponemes are widely considered to play an important role in periodontal disease etiology and pathogenesis (You et al., 2013). *Treponema putidum* is a novel treponeme isolated from lesions of periodontal disease and acute necrotizing ulcerative gingivitis (Wyss et al., 2004). The genus *Prevotella* is the largest genus in the phylum *Bacteroidetes* (Dewhirst et al., 2010) and contains species such as *P. intermedia* and *P. nigrescens*, members of the orange complex. Lesser known are organisms like *P. loeschii* which is, nonetheless, associated with periodontal disease. *P. loeschii* is known to produce propionic acid, a major metabolic player in the inflammatory process of gingival tissue (Al-Lahham et al., 2010). *Actinomyces* spp. are thought to play a significant role as early colonizers in the process of plaque formation and figure prominently in the microbial succession dogma of periodontal disease. The genus contains a total of 42 species, 20 of which are relevant for human health (Vielkind et al., 2015). *A. israelii* is part of the normal human oral flora and is frequently found in dental caries and at the gingival margins of individuals with poor oral hygiene (Desouches et al., 2019). It is the most common cause of actinomycosis, and has also been associated with periodontal disease (Vielkind et al., 2015). *Lautropia mirabilis* is a Gram-negative, motile, coccus bacteria that has been isolated from the human oral cavity (Gerner-Smidt et al., 1994). While *L. mirabilis* may be associated with some disease states (Ben Dekhil et al., 1997; Rossmann et al., 1998), it is currently primarily associated with periodontal health (Colombo et al., 2009; Balan et al., 2018; Papapanou et al., 2020). Finally, *Bacteroidaceae_[G-1] [G-1]_bacterium_HMT_272* is a novel, uncultivated, unnamed, and uncharacterized taxon. Little is known about this organism, however, it has been identified as the most prevalent phylotype in apical periodontitis lesions (Amaral et al., 2020).

In late pregnancy, four taxa were identified as being differentially abundant among women who spontaneously delivered early term. Women who delivered early term had lower abundances of *Catonella* sp.*_HMT_164, Tannerella* sp.*_HMT_286*, and *Campylobacter gracilis.* Both *Catonella* sp.*_HMT_164* and *Tannerella* sp.*_HMT_286* are uncultivated phylotypes. While these strains are not yet well characterized, we do know that other species within these genera are periopathogens. *Tannerella forsythia*, for example, belongs to the red complex and is known as a keystone periopathogen. *Catonella morbi*, the only named species within the genus, has been described as a putative pathogen (Wu et al., 2017). *Campylobacter gracilis* was one of the top 20 most abundant organisms across pregnancy in our cohort. *C. gracilis* belongs to the orange complex, known to closely precede colonization by the three dominant periopathogens of the red complex.

*Prevotella melaninogenica* was the only taxon identified to be more abundant among women who spontaneously delivered early term. *P. melaninogenica* is an oral commensal, gram-negative anaerobe that is closely related to periodontal disease (Ibrahim et al., 2017). *P. melaninogenica* has a pro-inflammatory effect that stimulates the release of inflammatory cytokines, suggesting that it may play an important role in promoting chronic inflammation (Larsen, 2017).

Several of the taxa identified to be differentially abundant between women who delivered early term *versus* full term in both early and late pregnancy were novel, uncultivated species suggesting the importance of looking beyond known pathogens to additional microbial players that may affect oral disease and birth outcomes. Many of the taxa identified to be differentially abundant between early term and full term women have strong known or suspected associations with periodontal disease. That most were elevated in women who delivered full term challenges the hypothetical association between periodontal disease (and periodontal disease-causing organisms) and adverse birth outcomes. Further investigation of these organisms, their role in periodontal disease and early term birth is required.

What our findings do reveal is that there are taxonomic shifts that are associated with early term birth. Two taxa are potential targets for future research. *Lautropia mirabilis* is a Gram-negative, motile, coccus bacteria that is known as an oral commensal (Gerner-Smidt et al., 1994). Studies have identified *L. mirabilis* as being associated with periodontal health (Colombo et al., 2009; Papapanou et al., 2020). In our study we identified that a depletion of this organism was associated with spontaneous early term birth suggesting that it may be a potential target of future investigation as an oral commensal associated with both oral and pregnancy health. *Prevotella melaninogenica* was the only taxa identified to be enriched in women who delivered early term. While not traditionally understood as a periopathogen, it has been identified to be highly abundant in the saliva of pregnant women (Balan et al., 2018), and enrichment of *Prevotella* spp. is associated with mucosal inflammation leading to systemic dissemination of inflammatory mediators, bacteria, and bacterial products, which in turn, may affect systemic outcomes (Larsen, 2017). Further investigation of this organism and its inflammatory effects on oral health and pregnancy are needed.

Spontaneous early term births are births that occur between 37 and 39 weeks. While infants born at this gestational age do not face the same risks as those born preterm (< 37 weeks), they do remain at increased risk for respiratory distress syndrome, transient tachypnea of the newborn, feeding difficulty, pneumonia, and hypothermia (Parikh et al., 2014). Although reasons for spontaneous early term birth are not fully understood, biological determinants may be at play including placental ischemia, diabetes, and poly- or oligohydramnios (Brown et al., 2015). To our knowledge, no studies have investigated the contribution of the oral microbiome to early term birth or tested the potential hypotheses that an oral microbiome shifted toward periodontal disease might contribute to early term labor. This study lays the groundwork and provides preliminary evidence for future targeted investigations testing this hypothetical association.

### 4.4 Limitations

Several limitations must be acknowledged. First, this was an exploratory study and was not fully powered to identify microbiome associations with preterm or early term birth. Second, although the Human Oral Microbiome Database offers a well-curated and up-to-date resource for 16S rRNA sequences, marker gene sequencing technology limits the resolution with which to identify taxa. Finally, the lack of periodontal disease clinical diagnosis limits our ability to interpret the clinical impact of the oral microbiome.

## 5 Conclusions

Despite these limitations, this exploratory study confirmed emerging findings that the diversity of the subgingival microbiome remains stable during pregnancy. While our study did not include clinical diagnosis of periodontal disease, our findings suggest that the subgingival microbiome of this group of pregnant women was shifted towards complexes associated with periodontal disease. Our exploration also identified periodontal disease associated taxa that differentiated the subgingival microbiome of women who delivered early term versus full-term, namely *Porphyromonas* sp.*_HMT_284, Catonella* sp.*_HMT_164, Peptostreptococcaceae_[XI][G-5] [XI][G-5]_saphenum, Johnsonella ignava, Actinomyces massiliensis* and *Cryptobacterium curtum*, suggesting a relationship between the subgingival microbiome and early term birth. Next step investigations should consider the incorporation of clinical assessment of the oral cavity; shotgun whole metagenome sequencing to allow identification of microbial taxa to the species, and even strain level; and further investigation into novel, uncultivated phylotypes which may play a role in the relationship between the subgingival microbiome in pregnancy and early term birth.

## Data Availability Statement

The datasets presented in this study can be found in online repositories. Repository: NCBI. Accession number: PRJNA811442. The link to the data can be found below: http://www.ncbi.nlm.nih.gov/bioproject/811442.

## Ethics Statement

The studies involving human participants were reviewed and approved by Emory University IRB. The patients/participants provided their written informed consent to participate in this study.

## Author Contributions

IY, EC, AD, NG, and VH contributed to the conception and design of the study. IY, RA, and HC contributed to the data curation. HC and IY performed the formal analysis. IY wrote the first draft of the manuscript. HC and RA wrote sections of the manuscript. All authors contributed to manuscript revision, read, and approved the submitted version.

## Funding

Support for this study came from NIH - National Institute of Nursing Research grant number R01NR014800 and NIH – National Institute of Nursing Research grant number K01NR016971.

## Conflict of Interest

The authors declare that the research was conducted in the absence of any commercial or financial relationships that could be construed as a potential conflict of interest.

## Publisher’s Note

All claims expressed in this article are solely those of the authors and do not necessarily represent those of their affiliated organizations, or those of the publisher, the editors and the reviewers. Any product that may be evaluated in this article, or claim that may be made by its manufacturer, is not guaranteed or endorsed by the publisher.
